# Perspectives on access and usage of assistive technology by people with intellectual disabilities in the Western Cape province of South Africa: Where to from here?

**DOI:** 10.4102/ajod.v10i0.767

**Published:** 2021-02-23

**Authors:** Fleur H. Boot, Callista Kahonde, John Dinsmore, Malcolm MacLachlan

**Affiliations:** 1 Department of Primary and Community Care, Faculty of Medicine, Radboud University Medical Centre, Nijmegen, The Netherlands; 2 Assisting Living and Learning (ALL) Institute, Department of Psychology, Maynooth University, Maynooth, Ireland; 3 Centre for Disability and Rehabilitation Studies, Faculty of Medicine and Health Sciences, Stellenbosch University, Cape Town, South Africa; 4 Centre for Practice and Healthcare Innovation, School of Nursing and Midwifery, Trinity College Dublin, Dublin, Ireland; 5 Olomouc University Social Health Institute (OUSHI), Palacky University, Olomouc, Czech Republic

**Keywords:** intellectual disability, assistive technology, access, health inequity, South Africa

## Abstract

**Background:**

Whilst assistive technology (AT) can play an important role to improve quality of life, health inequity regarding access to appropriate AT for people with intellectual disabilities (ID) is still very much present especially in low resource countries.

**Objectives:**

This study focused on exploring factors that influence access to and continued use of AT by people with ID in the Western Cape province of South Africa and to suggest potential implications of these findings and actions required to promote access to AT.

**Method:**

A qualitative approach was used to explore the experiences of people with ID and providers of AT. Face-to-face interviews with 20 adults with mild to profound ID, and 17 providers of AT were conducted and the data were analysed thematically.

**Results:**

People with ID within the study setting faced many challenges when trying to access AT and for those who managed to acquire AT, its continued usage was influenced by both personal characteristics of the user and environmental factors. Important factors that influence AT access and use for people with ID found in this study were (1) attitudes from the community, (2) knowledge and awareness to identify AT need and (3) AT training and instructions to support the user and care network.

**Conclusion:**

With the perspectives of both the providers and users of AT, this study identified priority factors, which could be addressed to improve AT access and use for people with ID in the Western Cape province.

## Introduction

Access to assistive technology (AT) has become an important topic on the global agenda towards implementation of the United Nations Convention on the Rights of Persons with Disabilities (UNCRPD), the Sustainable Development Goals (SDGs), and Universal Health Coverage (Tebbutt et al. 2016; UN 2006, 2015; World Health Organisation [WHO] 2016a). Assistive technology can play an important role to maintain or improve an individual’s functioning and health to enable people to live at home independently and to improve participation in society. Assistive technology ranges from low-tech products, such as glasses or pill organisers to high-tech products, such as motorised wheelchairs or communication software. Assistive technology can benefit a wide range of people, including people with disabilities, the ageing population and people with non-communicable diseases. The WHO stated that worldwide, only one out of 10 people has access to the AT they need, whilst it is expected that in 2030 more than 2 billion people will need at least one AT (WHO 2016a). In 2014, following the UNCRPD, the WHO launched the Global Cooperation on Assistive Technology (GATE) Programme to improve access to affordable and quality AT for everyone, all over the world (WHO 2014). Global Cooperation on Assistive Technology aims to address the different challenges in the field of AT policies and programmes, AT industry, AT service delivery and AT personnel, with a clear focus on the AT end-user whilst engaging with these challenges (WHO 2014, 2016a).

People with intellectual disabilities (ID) may benefit from access to AT. However, the use of AT for people with ID is still a neglected area in research and practice (Boot et al. 2017). The gap is even wider in low- and middle-income countries, where little is known regarding access and use of AT for people with ID even though the incidence of ID is significantly higher in low resource countries compared with high-income countries (Durkin 2002). People with ID have limitations in cognitive functioning and difficulties in coping with everyday tasks related to conceptual, social and practical skills (AAIDD 2013; The American Psychiatric Association 2013; WHO 2016b). Assistive technology may be useful in addressing these difficulties in cognitive and adaptive functioning and increasing independence and inclusion. In addition, people with ID have a high prevalence of comorbidities, which could be better managed with AT, such as sensory impairments, speech and language impairments or mobility disorders (Hatton & Emerson 2015; Jansen & Kingma-Thijsen 2011). However, the health needs of people with ID are often missed and there is a high rate of underdiagnoses of these comorbidities. For example, in a study by Meuwese-Jongejeugd et al. (2006), hearing impairment was found in 30% of the adults with ID, in half of the cases this hearing loss had not been diagnosed prior to the study (Meuwese-Jongejeugd et al. 2006).

People with ID often present health problems differently and may have difficulties in communicating their symptoms. They often depend on their care network to identify their health needs. In addition, people with ID are still a marginalised, devalued and stigmatised group and some of their health disparities are because of health inequities regarding access to secure health services and appropriate AT (Hatton & Emerson 2015).

South Africa is an upper middle-income country, according to the World Bank categorisation (The World Bank 2020). Although the exact prevalence of people with ID in South Africa remains unclear, it is expected to be around 3%, compared with 1% in high-income countries (Adnams 2010; McKenzie, McConkey & Adnams 2014). Specific epidemiological data on ID in South Africa is lacking and different methods and definitions are used to determine ID (Adnams 2010; Kleintjes et al. 2006; McKenzie, McConkey & Adnams 2013). It is likely that formal ID assessments do not often take place. McKenzie et al. (2013) found that people with ID in South Africa have limited access to healthcare and rehabilitation services. People with ID face profound difficulties when trying to secure South African human rights principles (Capri et al. 2018). Social services struggle to receive funding, resulting in organisations taking a more protective role instead of a social or human rights model approach to disability (McKenzie et al. 2014). Services and professionals also struggle to find context-relevant ID knowledge, because most ID research takes place in high-income countries. As research from high-income countries does not always fit the South African context and environment, there is a significant gap in indigenous research for people with ID, including research on ID and AT (McKenzie et al. 2013). Existing research on AT for persons with disabilities in South Africa and other parts of Africa has shown that AT for people with mobility impairments is given priority (Visagie et al. 2017). No existing studies were found specifically focusing on access to AT for persons with ID. Therefore, this study addressed the following research questions: Which factors influence access to and continued use of AT for people with ID in the Western Cape Province of South Africa? and how can the provision of AT for people with ID in South Africa be improved?

## Research methods and design

### Study design

This study adopted a qualitative research design using semi-structured face-to-face interviews within a phenomenological approach. Relatively few people with ID are able to read and write and to fill in written questionnaires. Therefore, the most appropriate method to gain personal views from people with ID is interviewing.

### Study participants

The participants were divided into two main groups: (1) adults with ID and (2) providers of AT.

Purposive sampling was used to recruit participants. Participants were approached through the network of the Centre for Disability and Rehabilitation Studies at Stellenbosch University in Cape Town, which consisted of psychiatric hospitals, care providers, disability persons’ organisations (DPOs), national umbrella bodies, parents’ advocacy groups, the Department of Social Development and local AT suppliers. For group 1 adults with ID, the management and gate-keepers of these different organisations were informed about the study and asked to identify 15 adults with ID willing to participate in an interview, either users or non-users of AT. Preference was given to select a range of individuals with different ages and levels of ID. The information leaflet and consent form were adjusted to the cognitive level of the participants (i.e. easy to read, larger font size, fewer words per row and the use of symbols) and were translated into two local languages in the Western Cape province, Afrikaans and Xhosa. If the participant was not able to give informed consent, his or her primary guardian gave informed consent. After informed consent, information on age, gender, care setting, level of ID, cause of ID (aetiology), medical history and indication or reason for having the AT was gathered. This information was provided by the parents or care staff. The participants were categorised into either mild-moderate or severe-profound ID by the researcher (first author) at the time of the interview, according to International Classification of Diseases (ICD)-10 classification (WHO 2016b). This categorisation was based on information available from the service providers and on the researcher’s experience as a specialist ID physician whilst communicating with participants and evaluating their understanding of the questions. Depending on the communication abilities of the participant, the interviews were conducted with the persons with ID themselves and/or their parents and care staff. Participants of group 2 providers of AT consisted of prescribers of AT (health professionals working with people with ID), local suppliers or retailers of AT, managers of disabled person’s organisations and government officials. Fifteen participants per group were expected to be sufficient to achieve saturation of the data from interviews (Van Schijndel-Speet et al. 2014). Achieving data saturation provides a comprehensive account for the specific groups interviewed in that resource setting. If the number of interviews did not achieve saturation of data, additional participants would be included.

### Data collection

The interviews took place in January and February 2018. The semi-structured interview guide focused on current use, needs, knowledge, awareness, access, customisation, funding, follow-up, social inclusion, stigma and policies of AT. All the interviews were conducted by the first author with the support of interpreters for participants who were non-English speaking. At the start of each interview, AT was defined using a booklet containing AT images to highlight the variety of AT. Assistive technology included any low- or high-tech product in the domains of vision, hearing, mobility, communication, cognition, environment and personal care. The questions were adjusted to the level of ID and the parents or care staff aided participants in understanding questions, which they found challenging. A copy of each interview guide is provided as Online Appendix 1 and 2. Interviews were conducted at a time and location convenient to the participant. The researcher explained the purpose of the interview and asked permission to use the audio recorder.

### Data analysis

The recorded interview data were first transcribed verbatim. The interviews conducted in local languages were transcribed and translated to English by translators who were proficient in the two languages. The technique of constant comparison analysis, as described by Elliott and Timulak (2005), was used to analyse the data. Firstly, participants’ responses were divided into meaning units. Meaning units are segments of the data that even if interpreted out of context would provide adequate information to the reader. Next, the meaning units were coded into categories that emerged from the meanings in the meaning units. The categories were subsequently organised into broad headings, or domains, to provide a conceptual framework for themes.

### Ethical consideration

This study is part of a larger cross-sectional study Global Access to Assistive Technology for People with Intellectual Disabilities (GATE-ID), for which ethical approval was obtained from the Health Policy & Management/Centre for Global Health Research Ethics Committee, Trinity College Dublin, Ireland (04/2017/01) and The Social Research Ethics Subcommittee, Maynooth University, Ireland (SRESC-2017-053). Ethical approval for this part of the study was granted by the Health Research Ethics Committee (HREC) of Stellenbosch University (HREC reference no.: N17/08/072) and the Western Cape Department of Health in Cape Town. The study adhered to the Declaration of Helsinki for research involving human subjects.

## Results

### Participant characteristics

In total, 37 participants were interviewed. Table 1 presents the participants’ characteristics of group 1 adults with ID (*n* = 20) and group 2 providers of AT (*n* = 17). The participants of group 1 were not professionally assessed for their intellectual functioning prior to the interview, so the researcher used the criteria described in the methods section to determine the level of ID. All adults with ID were accompanied by their caregiver (parent or care staff) during the interview to support them where needed. One adult was non-verbal and had a severe-profound ID in which case the caregiver answered all the questions for him.

**TABLE 1:** Participants characteristics.

Characteristics	People with ID (*n* = 20)	Providers (*n* = 17)
Age (mean)	40 years	Unknown
Female gender	10	14
**Level of ID**		
Mild – moderate	19	-
Severe – profound	1	-
**Aetiology**		
Acquired brain damage childhood	3	-
Perinatal asphyxia	2	-
Foetal alcohol syndrome (FAS)	1	-
Down syndrome	1	-
Goldenhar syndrome	1	-
Kabuki syndrome	1	-
Cerebellar ataxia	1	-
Spina Bifida	1	-
Ring chromosome 20	1	-
Unknown	8	-
**Racial composition**		
White	9	-
Coloured	2	-
Black	9	-
**Care setting**		
Centralised setting	10	-
With family	10	-
Semi-urban	5	-
Urban	15	-
**Work field**		
S&L therapist	-	2
Prosthetist and Orthotist	-	1
Occupational therapist	-	3
Physiotherapist	-	1
Medical doctor	-	1
Psychologist	-	2
Supplier of AT	-	2
Government representative	-	2
Management care provider	-	2
Management DPO	-	1
Urban	-	17

S&L, speech & language; DPO, disability persons’ organisation; ID, intellectual disabilities; AT, assistive technology.

### Assistive technology

Table 2 shows the current AT that participants from group 1 adults with ID were using.

**TABLE 2:** Current assistive technology in use by participants of group 1 adults with intellectual disabilities.

Code	Total no. of AT in use	Hearing	Vision	Communication	Mobility	Cognition	Environment and self-care
INT_ID_SA_001	2	0	0	1	1	0	0
INT_ID_SA_002	2	0	1	0	1	0	0
INT_ID_SA_003	5	0	1	1	1	0	2
INT_ID_SA_004	2	0	0	1	0	0	1
INT_ID_SA_005	1	0	0	1	0	0	0
INT_ID_SA_006	3	0	0	0	1	0	2
INT_ID_SA_007	1	0	0	1	0	0	0
INT_ID_SA_008	0	0	0	0	0	0	0
INT_ID_SA_009	0	0	0	0	0	0	0
INT_ID_SA_010	1	0	0	1	0	0	0
INT_ID_SA_011	4	0	1	1	2	0	0
INT_ID_SA_012	3	1	0	1	0	1	0
INT_ID_SA_013	0	0	0	0	0	0	0
INT_ID_SA_014	2	0	0	1	0	1	0
INT_ID_SA_015	9	2	2	3	0	0	2
INT_ID_SA_016	9	2	0	3	2	0	2
INT_ID_SA_017	3	2	0	1	0	0	0
INT_ID_SA_018	4	2	0	2	0	0	0
INT_ID_SA_019	5	1	1	3	0	0	0
INT_ID_SA_020	3	0	0	0	1	0	2
Average	3	1	0	1	0	0	1
**Total**	**59**	**10**	**6**	**21**	**9**	**2**	**11**

AT, assistive technology; ID, intellectual disabilities.

On average, participants used three AT products per person, ranging from zero to nine. The ATs most commonly used were in the domains of communication (cell phones) and environment or self-care (shower chairs). Products with AT to support hearing were mainly used by participants living with a care provider who serves individuals with hearing loss. The two participants with nine AT in use were also residents of this specialised care provider.

### Themes

Qualitative analysis of the data resulted in three main themes that are supported by findings from both groups: (1) stigma, (2) access to AT and (3) continued use of AT for people with ID. The results are presented here with the themes and domains as headings and subheadings, respectively. The domains are neither ordered in terms of importance nor do they imply any hierarchy. Each domain included meaning units from both group 1 adults with ID and group 2 providers of AT and the findings represent the perspectives of the participants of both groups. The findings are both facilitators and barriers related to the themes (*R* = Researcher; *P* = Participant).

#### Theme 1: Stigma

There were three domains generated for stigma: attitude towards ID, empowerment and advocacy, and shame.

**Attitude towards intellectual disabilities:** The majority of the shared examples of negative attitudes were not towards the AT but rather towards ID, and emerged from fear, poverty and lack of education according to the participants.

Participants shared their concern about the lack of knowledge within government, who they believed were not putting enough effort in supporting people with ID: ‘P: The issues around intellectual disabilities are almost always left off the table and forgotten’ (INT_PRO_SA_005 Government representative). Negative attitudes were also experienced from healthcare workers; people got refused at clinics and practitioners treated people with ID unequally:

‘P: I think they are just completely overlooked. If we have a person with ID and a child going to a community clinic and they both need a hearing aid, 10 to 1 the child gets in for assessment …. They are not going to refer the person to audiology and for hearing aids.’ (INT_PRO_SA_009, Occupational therapist)

Participants gave examples of negative attitudes from people in the community towards the AT they were using, for example:

‘R: Does it [*the wheelchair*] help you to make friends? P: No, not really. Because, people will look at you like, hhuuuhhh, who is this now. R: Is it difficult to make friends? P: Yes. Because most of them think if you’re in a wheelchair you’re not good enough.’ (INT_ID_SA_003, Adult with ID)

People with severe to profound ID were especially stigmatised when it came to AT; as if others had difficulties seeing beyond the cognitive limitations:

‘R: And his hearing, has that been tested? P: God. I don’t know, no. They don’t really go for, it’s only like mainly the high grades that already have glasses or stuff like that. R: You wouldn’t see people of his level to have those kind of products? P: No.’ (INT_ID_SA_020, Caregiver)

At community level, stigma was still very much seen as being culturally constructed:

‘P: There is still stigma in relation to ID in general. And a lot of it is culturally defined. In some cultures it’s viewed as being a curse. They are not allowed to be seen in public, it doesn’t matter what type of assistive device they have.’ (INT_PRO_SA_001, Government representative)

Carers mentioned that people with ID were exploited for criminal activities such as drugs and that they were often neglected by family members: ‘P: He is neglected really. The sister is getting his money [disability grant] and don’t buy him nothing’ (INT_ID_SA_008, Caregiver). In general, the experience reported was that people with ID were not seen as full citizens by people in the community and by the government:

‘P: The attitude that the patients who have ID and are wheelchair bound can’t really contribute that much to society anymore, is what they think. They choose not to fund that much because they are not getting anything back.’ (INT_PRO_SA_012, Physiotherapist)

**Empowerment and advocacy:** Participants mentioned that people with ID often do not go and ask for AT themselves and there was a lack of advocacy for people with ID:

‘P: A NGO that one can rely on, that you can go to. There are some, but that is more for your disabled person with a high IQ. It’s like you’re physically disabled with a high IQ, or a blind person with a high IQ, and deaf, for that there are many who take responsibility. But as soon as there is an IQ deficiency, it feels like people fall back.’ (INT_ID_SA_006, Caregiver)

In some cases, people with ID within a care facility were prohibited to have certain AT such as cell phones or iPads. However, in other cases, people with IDs were able to choose AT, such as glasses, themselves. Assistive technologies were enabling people with ID to be more independent:

‘R: Do you take your pills yourself, or does your mother help you with it? P: I take them myself. R: And if you wouldn’t have the pill organiser would you still be able to do it yourself? P: No, I wouldn’t.’ (INT_ID_SA_012, Adult with ID)

During AT assessment, it varied if people with ID were involved in the assessment process, to comply with the user’s needs and wishes and to see if the AT would fit the person correctly:

‘P: People often think that because of the compromised cognitive functioning they cannot consent or they can’t be involved in decision making processes. So they’re frequently not included. R: And is it the professional or the family that doesn’t include them? P: Both.’ (INT_PRO_SA_013, Psychologist)

Most of the health professionals did express that they included the person with ID within the decision-making process, although they might have to use a different approach:

‘P: Someone with an intellectual disability you have to make sure it’s tangible. You can’t be talking about abstract concepts, but if you just make sure your pictures are appropriate, the selection process is fine.’ (INT_PRO_SA_017, Occupational therapist)

**Shame:** Providers of AT mentioned that parents sometimes expressed shame towards AT and prohibited access to AT for their child:

‘R: Are people sometimes ashamed that they have to use the product? P: Yes, we see that a lot. A lot of the parents, although the child needs the product, they don’t actually want to buy it, because it’s almost like a confirmation of my child has a disability.’ (INT_PRO_SA_015, Supplier of AT)

Participants with ID themselves did not mention experiencing any shame regarding the use of AT: ‘R: How do you feel wearing the glasses? P: It’s like second nature. Basically it’s part of me. R: You’re not feeling ashamed for them? P: No, no’ (INT_ID_SA_015, Adult with ID).

#### Theme 2: Access to assistive technology

Five domains were generated for access to AT: Identifying AT need, assessment of AT need, financial, policy and systems (e.g. policies, resources and the organisation of AT services) and transport.

**Identifying assistive technology need:** ‘R: Do you think that people with ID could need some AT but don’t have it? P: Absolutely … hearing aids or spectacles, there is hundreds of people who need, but don’t have it. R: And do you know why? P: I think it’s either not thought of, someone with ID maybe we should check his hearing and vision.’ (INT_PRO_SA_006, Psychologist)

The carers who were present at the interviews could not think of any AT assessment the person with ID could benefit from. This indicates a lack of knowledge and awareness amongst carers regarding the health needs of people with ID and the range of AT that is available. The person with ID is often dependent on a carer or family member to identify the AT need:

‘P: When I was a girl of 11 years old, then I have been taken for an eye test. Our church did say that I needed to have glasses …. Then he [brother] said to my late mommy, take me then to have my eyes tested.’ (INT_ID_SA_002, Adult with ID)

Persons with severe to profound ID were seldom taken for AT assessment. Some professionals believed that people with ID living at care facilities would have better access to AT compared with people with ID living with families. Parents were not always aware that AT could make their life easier also. Both carers and professionals tended to focus on AT for ‘visible’ disabilities, such as mobility devices, and less on AT for communication or cognitive limitations. In addition, only a few speech and language therapists were available in public services. ‘P: Because communication is not as visible. Often they get physiotherapy first and mobility devices’ (INT_PRO_SA_016, Speech & Language therapist). To receive care and have access to AT for cognitive limitations, ID first needs to be identified. However, ID assessment rarely took place and ID often got confused with psychiatric diagnoses such as depression and psychoses:

‘R: Is ID often confused with psychiatric diagnoses like depression or psychoses? P: Yes. And the other way around. People with ID are often not diagnosed and treated for what they need.’ (INT_PRO_SA_006, Psychologist)

According to the health professionals from group 2, little training is provided on ID for health professionals during their studies to know which specific health needs are present for people with ID. Professionals indicated the need to receive training on AT for people with ID:

‘R: Who should be responsible for providing assistive products? P: I think we should all, as professionals, be able to do it, especially in South Africa. Because that person might be seen in a rural area, without access to an OT or physio or speechy, so I feel that the medical doctor should also be knowledgeable of all of these products.’ (INT_PRO_SA_008, Occupational therapist)

Next to the professional, it would be powerful if people with ID themselves could identify the need for AT and to know which AT could be beneficial to them. However, this wasn’t often the case:

‘P: No patient of mine has ever communicated that they need something. Only a few of them will say I can’t walk any longer, it’s too tired to walk to your programme from the ward, can I please get a wheelchair. But it’s those obvious assistive aids that they need.’ (INT_PRO_SA_008, Occupational therapist)

Pro-active healthcare assessments were not provided to people with ID, mainly because of a lack of (human) resources and a lack of knowledge:

‘P: We recently had a resident who ended up in the hospital because there was an injury to his eye, and he had cataract of the eye. We wouldn’t have known if he hadn’t ended up for something else.’ (INT_PRO_SA_010, Management care provider)

**Assessment of assistive technology need:** People need to be aware of available AT providers to have access to AT assessment. Assistive technology retailers and providers indicated that social media was helping carers to find them, but they ordinarily would not know where to go. Assistive technology providers cited advantages of community-based approaches to conduct AT assessment:

‘P: We had a lot of people scheduled for an appointment but they never came because they can’t afford it …. That is where they changed it to access the local clinic first, let’s do the assessment and find out what your needs are.’ (INT_PRO_SA_001, Government representative)

The importance of ID appropriate assessments was pointed out by several providers. However, some of the providers were not aware of assessments suitable to people with ID, for example, assessment possibilities for people with severe to profound ID in case of vision or hearing screening. Challenging behaviour could also be a barrier for providers to do an assessment. Providers were actively searching for training opportunities to develop their assessment skills for people with ID. In places where there was a lack of a variety of disciplines, the professional needed to be educated in several fields, for example, the occupational therapist was also playing the role of a physiotherapist and a speech and language (S&L) therapist.

Limited resources impacted on AT assessment:

‘P: We haven’t had a single person being send for a hearing test because quite frankly we don’t have the resources. We don’t have somebody we can send them to. It’s a big problem.’ (INT_PRO_SA_011, Management care provider)

Placement of new graduates is one solution to the problem of a lack of professionals in rural areas or public healthcare organisations in South Africa. A consequence of a lack of professionals is that people will buy AT randomly without any professional involved. ‘R: When the family got him the other wheelchair, how did they know which one to buy? P: They just bought a wheelchair, randomly’ (INT_ID_SA_020, Caregiver). The range of AT through the public health system was, in most cases, limited compared with the private sector. As a result, people would receive AT that wasn’t necessarily the AT they required. Another reason for people with ID to have a limited choice of AT would be because the professional did not have the time to train the person, whilst people with ID often need more (frequent) AT training compared with a person without ID.

Almost all participants from group 1 indicated they would ask support staff or a family member to know where to go, to make an appointment and accompany them for an AT assessment: ‘R: Do you remember who gave you the glasses? P: My mom made an appointment for me to go and get the glasses’ (INT_ID_SA_011, Adult with ID). Care facilities indicated it was not always easy to organise support to accompany the person to the AT assessment.

**Financial:** Funding was stated as a huge barrier for people to access AT. Participants agreed that the government should be (at least for those who cannot afford it) responsible to fund AT for people with ID. People with a disability grant were eligible to get AT from the tender list for free and some AT were indeed subsidised by government:

‘R: Is it expensive to go to the eye doctor? P: no. It’s free. R: And the glasses would you have to pay for that? P: no, it’s free at the hospital.’ (INT_ID_SA_009, Adult with ID)

However, there were limitations of public funding and the disability grant was not quite sufficient to afford AT that was not subsidised by the government:

‘P: They [local OT of public health system] can say this person needs a transfer board, but transfer boards are not in our system to give …. Incontinence products are only available for people over 60 years through the public health system.’ (INT_PRO_SA_002, Management DPO)

Also, the AT provided through the tender were more expensive:

‘P: What we find is that a lot of the products on the tender, … it’s ridiculous, it’s overpriced actually. The people who are supplying pushing the prices up high, because there are only a few suppliers. And they have been given the contract and they are part of the tender. It’s ridiculous expensive.’ (INT_PRO_SA_009, Occupational therapist)

If the government or medical aid did not fund the AT which is needed, people were left dependent on family resources. People with ID needed their own resources, whilst paid employment for people with ID was scarce:

‘R: Is it possible to get new ones [glasses] then? P: It is possible to get new ones, but I have to save up now the money, and that’s very, very difficult.’ (INT_ID_SA_011, Adult with ID)

Participants tried to get funding to buy AT through charities, NGOs, fundraisers and corporate sponsorship or get access to recycled AT, occasionally available from care facilities.

Participants indicated the advantage of better networking and intersectoral collaboration to fund AT through the groups listed here.

**Policy and systems:** Most participants indicated the need and advantages for having an AT human rights policy in place to set standards and to push organisations to increase access and provide services for AT:

‘R: Do you think there is a need for a national AT policy programme? P: Definitely. Because it gives you more leverage to hold into account. We don’t even have a disability act in this country. It would also regulate what we do as a non-profit organisation. Legislation is important.’ (INT_PRO_SA_002, Management DPO)

Participants indicated that services for people with ID were quite disjointed throughout their lives. It would help if only one access or contact point for AT provision and maintenance was created within the government despite the department referred to or the age of the user. Access to AT was easier for children with ID going to special schools, where AT was provided by the school, compared with adults with ID. Also, people attending sheltered workplaces would be more familiar with AT. Both the schools and the workplaces would refer people with ID to AT providers:

‘P: I think that access to education that helps a lot. Once they are in some kind of school, then there is the possibility to get the assessment and getting to know about it [AT].’ (INT_PRO_SA_014, Speech & Language therapist)

However, (high-tech) communication devices were not available through education systems. It was reported that the tender list developed by the government for public funding of AT had limited AT available for communication and cognition. Overall there was a lack of knowledge and awareness around AT and its benefits within the government: ‘P: They don’t realise, especially at a government level, that things like communication boards are better than talking in some cases, more effective’ (INT_PRO_SA_015, Occupational therapist). Ten years after ratifying, the UNCRPD participants stated that implementation was poor and ID was not really a priority for government: ‘P: Signing it seemed like a great idea. They signed it and then they thought about it. We acted without thinking … and no one knows what to do’ (INT_PRO_SA_005, Government representative).

**Transport:** Accessibility of the outdoor infrastructure and (public) transport vehicles was limited. It helped if AT providers were close by, if providers did on-site visits or if AT was delivered locally and people did not have to travel far. For rural areas, it was important that local clinics were established to limit traveling. Some people still had to travel far to get to their AT provider:

‘R: Do people have to travel a lot to get to places for assessment? P: A lot! A lot. I mean, say for example on communication, there is only one real centre in the country that does like detailed assessment. In Pretoria. There might be some therapists in Cape Town, but it’s not that common.’ (INT_PRO_SA_014, Speech & Language therapist)

Transport could be a huge barrier because of costs. Public transport is very poorly regulated and families or care facilities would need their own resources to cater for transport. Because people with ID often need someone to accompany them to or during the AT assessment, transport costs such as a taxi ride can be a double expense: ‘P: The van [of my mother] broke. So we don’t have transport to transport me. So I have to wait till she fixed the van to take me’ (INT_ID_SA_011, Adult with ID). There were a few governmental initiatives to fund transportation:

‘P: There is a system in the Western Cape which is called health net, which is for free for public patients to come to hospitals …. P: It’s like a bus or small mini bus service. But it’s not really competent, it’s not really well run and it’s completely fully booked.’ (INT_PRO_SA_003, Prosthetist and Orthotist)

One retailer mentioned a solution to overcome transport issues:

‘P: If we can do sort of satellite type systems, where if we at least trained some people in a specific region or district, and then they can then sort of start the process. And if they need you to come and consult, we can.’ (INT_PRO_SA_017, Supplier of AT)

#### Theme 3: Continued usage

Data coding developed into five domains for continued use of AT: acceptance, context, follow-up and maintenance, impact and support.

**Acceptance:** Professionals indicated the importance for the user to feel comfortable with the AT in order to accept it and use it daily. Sometimes alternatives for AT were preferred by the user or the carer. If the new AT was not accepted by the family or the community, the AT was not used.

Challenging behaviour could also be a barrier to accept and use AT:

‘P: We’ve tried using pictures and things like that [for communication], but it’s challenging because of challenging behaviour. We’ve got a lot of people who take everything down what they see and throw it away.’ (INT_PRO_SA_010, Management care provider)

Carers and users sometimes struggled with new AT, which resulted in abandonment, especially when the use of AT was very time consuming:

‘P: With the lower functioning patients we have a big challenge with that in terms of getting used to the device. For example the “B” spoon, just because the fact it looks different and it is painted in a funny way, they are not interested in it.’ (INT_PRO_SA_004, Occupational therapist)

**Context:** To ensure continuous use of AT, customisation to the user’s needs was highly important. Occupational therapists played an important role in care facilities to customise AT with the little resources they had. For people living with families, customisation was expected to be less:

‘R: Are there any footrests that came with the wheelchair? P: there was but it has no value for her. R: aren’t they the right height? P: they are too low and you know, she’s very short if you look at where her feet are, so I have to, those things have to sit around here to really get it lifted. So we don’t ever use the footrests.’ (INT_ID_SA_006, Caregiver)

In addition, customisation of the AT to the context of the user was extremely important to ensure feasibility of AT. Some living environments, such as shacks, were too small to fit large AT or did not have electricity for AT. Mobility products needed to be adapted to the rural roads or glasses needed to be customised for challenging behaviour.

**Follow-up and maintenance:** There were variations in the participants’ accounts regarding implementation of structural follow-up. A lack of staff would prohibit follow-up sometimes, and AT provided by the government directly to the person did not include a follow-up programme. Some of the users were aware of going for check-ups, others did not feel the need. Most users depended on their carers to identify the need for follow-up and maintenance to know where to go and to make an appointment:

‘P: Unfortunately because these patients can’t phone they rely on others. That’s a really good point why they are not coming back …. But you also don’t want them to come back for nothing [with all the expenses to get there] and say it’s all ok.’ (INT_PRO_SA_003, Prosthetist and Orthotist)

Transport and funding could be a major barrier to ensure follow-up and maintenance:

‘R: Where would you go to, to check it? P: Cape Town. But then I would be a while without it. Because there is not usually somebody that goes to Cape Town regularly. Then I must wait.’ (INT_ID_SA_016, Adult with ID)

Often people would go to a non-professional to repair the AT:

‘R: And if it breaks where do you go? P: Sometimes I tell my mom. Maybe she can phone her boss, a friend maybe who can help me to fix it … sometimes there is a guy on the ground that also tries wherever he can.’ (INT_ID_SA_016, Adult with ID)

Facilitators to ensure follow-up, were reminders sent by the provider, maintenance identified by the user him- or her-self and sometimes a proactive community approach:

‘P: We have community rehabilitation workers, under the supervision of the clinics who also go in to see if everything is ok. We have outreaches who do the follow-up as well.’ (INT_PRO_SA_001, Government representative)

**Impact:** It was stated that it was important to make AT part of the daily routine. It helped if the user enjoyed using their AT. When a user was aware of the benefit the AT had for her or him, continuous use was more likely to occur: ‘P: I feel I can see better when I have the glasses on. Without them I feel lost’ (INT_ID_SA_002, Adult with ID). Mobility devices were helping the users to undertake physical activities, which they would not be able to do without it. Most users explained that the AT they were using was making them feel happy. One participant did not like the pill organiser because it was too complicated to use. Others struggled when using new AT, but were feeling better about it over time. Especially the use of a mobile phone made a lot of participants feel very happy: ‘P: For their self-confidence the mobile phone is wonderful’ (INT_ID_SA_018, Caregiver). Mobile phones enabled them to have social contacts with friends and family:

‘R: Why would you like a smartphone? Participant A: To communicate with other people. That also have my problem. R: And can you explain what your problem is? P: Yes, I can’t read and write.’ (INT_ID_SA_013, Adult with ID)

**Support:** Some users were fully dependent on carers supporting their daily use of AT. Others could use the AT mostly independently but would need a (verbal) reminder to pick-up the AT and use it.

‘R: And now if you want to use it [cell phone], can you do it on your own? Or do you need help? P: sometimes on my own, sometimes with some help. R: And who do you ask for help? P: My brother’s son.’ (INT_ID_SA_010, Adult with ID)

Barriers to individual support in care facilities were linked to low staff to client ratios, high staff turnover or a lack of staff. Independent use of AT was a huge facilitator to ensure daily use of the AT. Users did need some type of training or instructions to do this successfully. However, training was not always feasible:

‘P: Getting somebody glasses. You know that the pair of glasses will probably significantly improve the quality of life … But we don’t have the resources to support that person in terms of teaching how to look after it. So you can’t give that support. So in the end you just don’t issue.’ (INT_PRO_SA_011, Management care provider)

The different languages within South Africa also made it more complicated to ensure adequate training:

‘P: I don’t think it’s only that they don’t necessarily know they have forgotten … but it’s also the people who care for them, they might also forget. A very important issue in this is language. So many of the people we work with, English is not their first language. That has to be considered in how things are explained.’ (INT_PRO_SA_013, Psychologist)

Some providers would not provide AT if carers were not able to support the user and some providers adapted their training to the level of ID. Occasionally, users or carers never received any training or instructions with the AT:

‘P: If it’s one of our in-patients, a nurse will accompany them to the hospital. But they are not informed by the prosthetist or whomever how to coach and how to help. So they come back and the person stops using the device, and then the nurses ask why are you not using the device, that’s the end of it.’ (INT_PRO_SA_006, Psychologist)

Peer learning helped people with ID to understand how to use their AT:

‘R: Is it easy to use the cell phone? P: Yes it’s easy. I know how to work with smartphones … I learned from my friends, without reading or anything. But I can’t read or type. I can type in Afrikaans, like my name or something, but other words I can’t.’ (INT_ID_SA_003, Adult with ID)

## Discussion

This qualitative research study in the Western Cape Province of South Africa presents an overview of factors influencing AT access and use for people with ID. These factors can be used to guide government and health professionals to improve the current situation of AT-related health inequalities and limitations for participation in society for people with ID in the study setting. Assistive technology can play an important role towards realising the SDGs and UNCRPD, which South Africa ratified in 2007. The findings raise concerns about gaps in access and usage of AT by people with ID and critical factors impacting these, such as attitudes towards ID and AT, knowledge and awareness to identify AT need and AT training and instructions to support the user and care network.

A first potential action suggested by the authors following these findings is providing training and education on ID and stigma. It is known that there is a lack of services and resources allocated to the care of persons with ID globally (WHO 2007). Part of this is because of stigma and a lack of knowledge and awareness on ID and health needs at every level of society.

People with ID are one of the most excluded and marginalised groups in society (Ali et al. 2012; Hatton & Emerson 2015). The majority of people with ID in South Africa live with family who need to cope with stigma often present within their society (McKenzie & McConkey 2016; McKenzie et al. 2014). There is a high level of public stigma in South Africa towards ID mostly because of a lack of understanding and fear of the disability. Education might help to reduce fear and it can also clear the confusion between mental health problems and ID. In South Africa, it is common to refer to ID as a mental illness or psychiatric disorder (Mkabile & Swartz 2020). Participants stated that people from society and professionals often did not seem to know the difference between certain psychiatric diagnosis, such as psychosis and ID. The fact that some of the residential facilities for persons with ID are housed within psychiatric hospitals, and not ID care facilities or other alternatives which are not directly linked to a hospital or psychiatry based, support these findings and needs to be questioned. The organisation of services for people with disabilities in South Africa still reflects a medical model approach where people with ID are regarded as patients needing treatment, instead of viewing them as members of the community, where they can be supported to acquire life skills similar to non-disabled peers. This strong medical model approach may prohibit individual AT user empowerment. People with ID often do not have the opportunity to visit professionals themselves and request AT. The extent to which the views of people with ID themselves were included during AT assessments also varied. Self-advocacy for people with ID is one of the key areas significantly lagging, which could help to raise awareness of the importance of AT assessment and use. High standard, context appropriate and ID-specific training programmes are limited and should be developed for the South African context. For example, these training programmes need to be mindful of indigenous knowledge to understand the social script around AT in South Africa.

For access, the most frequent response was related to the importance of identifying AT need, especially by the user themselves. People tend to focus on visible disabilities, whilst other needs are often missed or not thought of such as AT to improve communication. A second potential action suggested by the authors is creating peer learning initiatives. Other research has shown that peer learning can play an important role for people with ID to realise which AT they could use and how it may benefit them (Boot, MacLachlan & Dinsmore 2019). People with ID often need to see examples of other people with similar disabilities using AT in order to realise that AT is available for them and they are capable of using it (Boot et al. 2019). As a result of a lack of resources and professionals to create AT awareness especially in the rural areas, peer learning initiatives may be a much better option. Being in a school or workshop is also an advantage in terms of identifying AT need. Hence, an important action will be encouraging families of persons with ID to access services and for responsible authorities to ensure availability of these services. Limited infrastructure and transport options were seen as a huge barrier to access AT for people with ID by the participants. Although transport and accessibility could apply to people with all types of disabilities, it was presented as a specific barrier to people with ID in relation to stigma. People with ID and their care network shared experiences of being refused access to transport options.

The main factor to ensure continued use is providing AT support, including AT training and instructions. If AT is provided without any training or instructions to the user and their care network, it is likely that they will not use it. In addition, AT-use will be more sustainable amongst those who are able to independently use their AT. For people with ID, training and instructions need to be available over time to ensure they remember how to use their AT correctly. Assistive technology training can be included within a peer learning environment as mentioned here. Self-help groups amongst people with ID and their families could offer possibilities for recurring, accessible peer learning initiatives.

The number of AT in use by the participants of this study varied greatly from zero to nine per person, depending on the living environment and level of ID. Those people living at a specialised care provider were using multiple AT. These participants had multiple disabilities, which required specialised care with knowledgeable professionals providing multiple AT. Those participants who did not use AT or only a small number of AT were primarily not aware of available AT, which could be beneficial to them. One critical area related to limited use of AT is lack of coordinated collaboration amongst different service providers, and the end-users and their families to share resources and knowledge on ID and AT need. To facilitate participation and inclusion for people with disabilities in South Africa, it is crucial that effective communication and collaboration between service providers and people with disabilities and their families is established (Muller, Ned & Duvenage 2015). Presently, knowledge is disjointed that results in some people with ID not getting the necessary assessments and prescription of AT.

Proactive AT assessments did not take place, especially for those with severe to profound ID. Whilst it is known that the prevalence of certain comorbidities is related to the severity of ID, such as hearing or visual impairments, participants stated that it was often not thought of, to assess people with severe ID on non-visible impairments such as these. The Western Cape policy framework for services to people with ID also primarily focuses on AT supporting visible impairments such as mobility and seating (Western Cape Government 2015). Providers did not always have the skills to conduct an assessment with people with ID. Some of the providers mentioned that they were not aware of assessments suitable to people with ID, for example, vision or hearing screening assessments for people with severe to profound ID. Challenging behaviour could also be a barrier for providers to conduct an assessment. However, providers were keen to search for training opportunities to develop their assessment skills for people with ID. These findings show that ID is neglected in the training of service providers and considerations need to be made to introduce ID specific concepts in the training of rehabilitation and other relevant professionals and continued professional development opportunities in this area.

South Africa has a national rehabilitation policy (South Africa Department of Health 2000) to improve accessibility to all rehabilitation services based on the principles of community-based rehabilitation (WHO 2010). Currently, community-based workers assist with follow-up but the process is not structured and coordinated as shown by the findings. A clear understanding of the need for AT by persons with ID and its impact in improving their quality of life and independence is imperative for providers of community-based services. Also at local policy level, there is a Western Cape policy framework for services to people with ID (Western Cape Government 2015) and a South African national guideline on provision of assistive devices in the public health sector (South Africa Department of Health 2003), which should guide the process. However, participants clearly indicated that policy implementation was lacking and services for people with ID were quite disjointed. Further research to understand the reasons why these policies are not being implemented as they should is recommended.

## Conclusion and way forward

Research within the field of ID and AT in the African context is rare. Although this was a small-scale study focusing on one province of South Africa, the findings highlight poor access and use of AT by people with ID in this part of the country, even though research elsewhere has shown that people with ID can greatly benefit from AT (Boot et al. 2017; Owuor, Larkan & MacLachlan 2017). With the perspectives of both the providers of AT and the users of AT, this study presents an overview and identifies priority areas that could be addressed to improve AT access and use for people with ID in the Western Cape province. To understand which actions can contribute most in different contexts, more research is needed and particularly research that foregrounds the views and experiences of people with ID themselves, as well as service providers. Lastly, the current growing possibilities of AT and the global trend of digitalisation calls for consideration of how AT is being used by people with ID, so that they are not left behind. Assistive technology in this aspect can be viewed broader than the external products and services, to include aspects such as universal design.

## References

[CIT0001] Aaidd, 2013, *Definition of intellectual disability*, viewed 11 February 2021, from https://aaidd.org/intellectual-disability/definition#.V_UMUIWcHIU

[CIT0002] Adnams, C.M., 2010, ‘Perspectives of intellectual disability in South Africa: Epidemiology, policy, services for children and adults’, *Current Opinion in Psychiatry* 23(5), 436–440. 10.1097/YCO.0b013e32833cfc2d 20683179

[CIT0003] Ali, A., Hassiotis, A., Strydom, A. & King, M., 2012, ‘Self stigma in people with intellectual disabilities and courtesy stigma in family carers: A systematic review’, *Research in Developmental Disabilities* 33(6), 2122–2140. 10.1016/j.ridd.2012.06.013 22784823

[CIT0004] Boot, F.H., Dinsmore, J., Khasnabis, C. & MacLachlan, M., 2017, ‘Intellectual disability and assistive technology: Opening the GATE wider’, *Frontiers in Public Health* 5, 10. 10.3389/fpubh.2017.00010 28275593 PMC5319964

[CIT0005] Boot, F.H., MacLachlan, M. & Dinsmore, J., 2019, ‘Are there differences in factors influencing access and continues use of assistive products for people with intellectual disabilities living in group homes?’, *Disability and Rehabilitation: Assistive Technology* 15(2), 173–182. 10.1080/17483107.2018.1550116 30689464

[CIT0006] Capri, C., Abrahams, L., McKenzie, J., Coetzee, O., Mkabile, S., Saptouw, M. et al., 2018, ‘Intellectual disability rights and inclusive citizenship in South Africa: What can a scoping review tell us?’, *African Journal of Disability* 7, a396. 10.4102/ajod.v7i0.396 PMC5968870 29850438

[CIT0007] Durkin, M., 2002, ‘The epidemiology of developmental disabilities in low-income countries’, *Mental Retardation and Developmental Disabilities Research Reviews* 8(3), 206–211. 10.1002/mrdd.10039 12216065

[CIT0008] Elliott, R. & Timulak, L., 2005, ‘Descriptive and interpretive approaches to qualitative research’, in J. Miles & P. Gilbert (eds.), *A handbook of research methods for clinical and health psychology*, pp. 147–160, Oxford University Press, New York, NY.

[CIT0009] Hatton, C. & Emerson, E., 2015, *International review of research in developmental disabilities – Health disparities and intellectual disabilities*, Academic Press Elsevier, Waltham.

[CIT0010] Jansen, A. & Kingma-Thijsen, J., 2011, *Searching for physical explanations for challenging behaviour in people with an intellectual disability* (in Dutch), p. 191, Centrum voor Consultatie en Expertise (CCE), Utrecht.

[CIT0011] Kleintjes, S., Flisher, A.J., Fick, M., Railoun, A., Lund, C., Molteno, C. et al., 2006, ‘The prevalence of mental disorders among children, adolescents and adults in the Western Cape, South Africa’, *African Journal of Psychiatry* 9(3), 157–160. 10.4314/ajpsy.v9i3.30217

[CIT0012] McKenzie, J. & McConkey, R., 2016, ‘Caring for adults with intellectual disability: The perspectives of family carers in South Africa’, *Journal of Applied Research in Intellectual Disabilities* 29(6), 531–541. 10.1111/jar.12209 26358760

[CIT0013] McKenzie, J., McConkey, R. & Adnams, C., 2014, ‘Residential facilities for adults with intellectual disability in a developing country: A case study from South Africa’, *Journal of Intellectual & Developmental Disability* 39(1), 45–54. 10.3109/13668250.2013.865157

[CIT0014] McKenzie, J.A., McConkey, R. & Adnams, C., 2013, ‘Intellectual disability in Africa: Implications for research and service development’, *Disability and Rehabilitation* 35(20), 1750–1755. 10.3109/09638288.2012.751461 23350758

[CIT0015] Meuwese-Jongejeugd, A., Vink, M., Van Zanten, B., Verschuure, H., Eichhorn, E., Koopman, D. et al., 2006, ‘Prevalence of hearing loss in 1598 adults with an intellectual disability: Cross-sectional population based study’, *International Journal of Audiology* 45(11), 660–669. 10.1080/14992020600920812 17118908

[CIT0016] Mkabile, S. & Swartz, L., 2020, ‘Caregivers’ and parents’ explanatory models of intellectual disability in Khayelitsha, Cape Town, South Africa’, *Journal of Applied Research in Intellectual Disabilities* 33(5), 1026–1037. 10.1111/jar.12725 32232922

[CIT0017] Muller, J., Ned, L. & Duvenage, M., 2015, ‘A coordinated collaborative response to rehabilitation needs of persons with disabilities’, *Archives of Physical Medicine and Rehabilitation* 2(7), 1056.

[CIT0018] Owuor, J., Larkan, F. & MacLachlan, M., 2017, ‘Leaving no-one behind: Using assistive technology to enhance community living for people with intellectual disability’, *Disability and Rehabilitation: Assistive Technology* 12(5), 426–428. 10.1080/17483107.2017.1312572 28453333

[CIT0019] South Africa Department of Health, 2000, *Rehabilitation for all: National rehabilitation policy*, viewed 11 February 2021, from https://www.mindbank.info/item/3319

[CIT0020] South Africa Department of Health, 2003, *Standardisation of provision of mobility assistive devices in South Africa: A guideline for use in the public sector*, viewed 11 February 2021, from http://uhambofoundation.org.za/new_wp/wp-content/uploads/2016/06/standardisation_of_provision_of_assistive_devices_in_south_.pdf

[CIT0021] Tebbutt, E., Brodmann, R., Borg, J., MacLachlan, M., Khasnabis, C. & Horvath, R., 2016, ‘Assistive products and the sustainable development goals (SDGs)’, *Globalization and Health* 12, 79. 10.1186/s12992-016-0220-6 27899117 PMC5129191

[CIT0022] The American Psychiatric Association, 2013, *DSM-5*, American Psychiatric Publishing, Arlington, VA.

[CIT0023] The World Bank, 2020, *World Bank data*, viewed 11 February 2021, from https://data.worldbank.org

[CIT0024] UN, 2006, *Convention on the rights of persons with disabilities (CRPD)*, viewed 11 February 2021, from https://www.un.org/development/desa/disabilities/convention-on-the-rights-of-persons-with-disabilities.html 10.1515/9783110208856.203 18348362

[CIT0025] UN, 2015, *Sustainable development goals*, viewed 11 February 2021, from http://www.un.org/sustainabledevelopment/sustainable-development-goals/

[CIT0026] Van Schijndel-Speet, M., Evenhuis, H.M., Van Wijck, R., Van Empelen, P. & Echteld, M.A., 2014, ‘Facilitators and barriers to physical activity as perceived by older adults with intellectual disability’, *Intellectual and Development Disabilities* 52(3), 175–186. 10.1352/1934-9556-52.3.175 24937743

[CIT0027] Visagie, S., Eide, A.H., Mannan, H., Schneider, M., Swartz, L., Mji, G. et al., 2017, ‘A description of assistive technology sources, services and outcomes of use in a number of African settings’, *Disability and Rehabilitation: Assistive Technology* 12(7), 705–712. 10.1080/17483107.2016.1244293 27882821

[CIT0028] Western Cape Government, 2015, *Policy framework: Services to persons with intellectual disability*, viewed March 2020, from https://www.westerncape.gov.za/assets/departments/social-development/2015_policy_framework_-_services_to_persons_with_intellectial_disability_1.pdf

[CIT0029] WHO, 2007, *Atlas: Global resources for persons with intellectual disabilities*, WHO Press, Geneva, viewed 11 February 2021, from http://www.who.int/mental_health/evidence/atlas/atlas_intellectual_disabilities_2007/en/10.1590/s0036-36342008000800009 18470344

[CIT0030] WHO, 2010, *Community-based rehabilitation guidelines*, viewed 11 February 2021, from https://apps.who.int/iris/handle/10665/44405

[CIT0031] WHO, 2014, *Global cooperation on assistive technology (GATE)*, viewed 11 February 2021, from https://www.who.int/phi/implementation/assistive_technology/phi_gate/en/10.1080/17483107.2018.1467971 29741127

[CIT0032] WHO, 2016a, *Assistive technology fact sheet*, viewed 11 February 2021, from http://www.who.int/mediacentre/factsheets/assistive-technology/en/

[CIT0033] WHO, 2016b, *ICD-10 mental retardation*, viewed 11 February 2021, from http://apps.who.int/classifications/icd10/browse/2016/en#/F70-F79

